# Functional and Prognostic Implications of the Main Pulmonary Artery Diameter to Aorta Diameter Ratio from Chest Computed Tomography in Korean COPD Patients

**DOI:** 10.1371/journal.pone.0154584

**Published:** 2016-05-06

**Authors:** Kyung Soo Chung, Young Sam Kim, Se Kyu Kim, Ha Yan Kim, Sang Min Lee, Joon Beom Seo, Yeon Mok Oh, Ji Ye Jung, Sang-Do Lee, Korean Obstructive Lung Disease study group

**Affiliations:** 1 Division of Pulmonology, Department of Internal Medicine, Institute of Chest Disease, Severance Hospital, Yonsei University College of Medicine, Seoul, Republic of Korea; 2 Biostatistics Collaboration Unit, Yonsei University College of Medicine, Seoul, Republic of Korea; 3 Department of Radiology & Research Institute of Radiology, Asan Medical Center, University of Ulsan College of Medicine, Seoul, Republic of Korea; 4 Department of Pulmonary and Critical Care Medicine and Clinical Research Center for Chronic Obstructive Airway Diseases, Asan Medical Center, University of Ulsan College of Medicine, Seoul, Republic of Korea; University of Palermo, ITALY

## Abstract

**Background:**

The ratio of the diameter of the main pulmonary artery (mPA) to the diameter of the aorta (Ao) on chest computed tomography is associated with diverse clinical conditions. Herein, we determined the functional and prognostic implications of the mPA/Ao ratio in Korean chronic obstructive pulmonary disease (COPD) patients.

**Methods:**

The study population comprised 226 chronic obstructive pulmonary disease patients from the Korean Obstructive Lung Disease cohort who underwent chest computed tomography. We analyzed the relationships between the clinical characteristics, including pulmonary function, echocardiography findings, St. George's Respiratory Questionnaire, 6-minute walking (6MW) distance, and exacerbation with the mPA, Ao, and mPA/Ao ratio.

**Results:**

The mean age was 65.8 years, and 219 (96.9%) patients were male. The mean FEV_1_% predicted and FEV_1_/FVC ratio were 61.2% and 47.3%, respectively. The mean mPA and Ao were 23.7 and 36.4 mm, respectively, and the mPA/Ao ratio was 0.66. The mPA/Ao ratio correlated negatively with the 6MW distance (G = -0.133, *P* = 0.025) and positively with the right ventricular pressure (G = 0.323, *P* = 0.001). After adjustment for potential confounders, the mPA/Ao ratio was significantly associated with 6MW distance (*β* = -107.7, *P* = 0.017). Moreover, an mPA/Ao ratio >0.8 was a significant predictor of exacerbation at the 1-year (odds ratio 2.12, 95% confidence interval 1.27–3.52) and 3-year follow-ups (odds ratio 2.04, 95% confidence interval 1.42–2.90).

**Conclusions:**

The mPA/Ao ratio is an independent predictor of exercise capacity and an mPA/Ao ratio >0.8 is a significant risk factor of COPD exacerbation.

## Introduction

Chronic obstructive pulmonary disease (COPD) is characterized by progressive, irreversible airflow limitations, resulting in disabling respiratory symptoms and devastating comorbidities. Pulmonary hypertension (PH) occurs in severe COPD due to various mechanisms, including hypoxic vasoconstriction, pulmonary hyperinflation with increased intrathoracic pressure, and loss of pulmonary vascular capacity due to parenchymal destruction.[1–3] However, similar changes can be recognized in mild COPD, suggesting that systemic vascular alterations such as endothelial dysfunction are closely related with PH.[4,5]

PH is an important predictor of low exercise capacity, exacerbations, and mortality in COPD.[6–8] However, PH in COPD is rarely diagnosed using right heart catheterization as the gold standard, because of its relative invasiveness. Doppler echocardiography is a noninvasive means of estimating systolic pulmonary artery pressure and diagnosing significant PH. Moreover, high negative predictive value may make echocardiography as one of the screening modalities for PH in patients with severe emphysema. However, the hyperinflated lungs in COPD may decrease its sensitivity.[9,10]

The ratio of the main pulmonary artery diameter (mPA) to the aorta diameter (Ao) on chest computed tomography (CT) correlates with the invasive measures of pulmonary artery pressure and is one useful method to detect PH.[11,12] Furthermore, relative pulmonary artery enlargement (mPA/Ao ratio >1), as measured by chest CT, is associated with an increased risk of COPD exacerbation and hospitalization.[13]

However, other clinical implications of the mPA/Ao ratio in COPD remain largely unknown. Hence, we investigated the functional and prognostic implications of the mPA/Ao ratio in a cohort of Korean COPD patients.

## Materials and Methods

### Study population

Two hundred twenty-six COPD patients from the Korean Obstructive Lung Disease (KOLD) cohort, in which patients with COPD or asthma were enrolled from pulmonary clinics in 17 hospitals in South Korea from June 2005 to December 2012, were included in the present study. The inclusion criteria were as follows: post-bronchodilator ratio of forced expiratory volume in 1 second to forced vital capacity (FEV_1_/FVC) <0.7, age over 40 years, smoking history of 10 or more pack-years, no or minimal abnormality on chest radiography, and available chest CT data. Of the 226 participants, 205 (90.7%) and 171 (75.7%) had completed 1 and 3 years of follow-up, respectively. This study was conducted in accordance with the amended Declaration of Helsinki. The Institutional Review Boards of the 17 hospitals included in the KOLD cohort approved the protocol and written informed consent was obtained from all patients: Asan Medical Center IRB (2012–0226), Hanyang University Guri Hospital IRB (2012–016), Inje University Ilsan Paik Hospital IRB (05–06), Bundangcha Hospital IRB (2005–017), Kangbook Samsung Medical Center IRB (2005–19), Ewha Womans University Mokdong Hospital IRB (106–02), Kangwon National University Hospital IRB (05–01), Korea University Anam Hospital IRB (254), Seoul National University Hospital IRB (H-0505-148-013), Seoul National University Bundang Hospital IRB (B-05081023-009), Hallym University Medical Center IRB (2005–9), Konkuk University Medical Center IRB (KUH 1010210), Ajou University Hospital IRB (10–237), National Medical Center IRB (CM-KOLD-1109), The Catholic University of Korea Seoul St. Mary's Hospital IRB (KC-11-OIME-0668), The Catholic University of Korea Yeouido St. Mary's Hospital IRB (SC12-RIMI-0078), Severance Hospital IRB (4-2006-0101).

### Clinical characteristics of COPD

Data on demographics, smoking status, underlying diseases, pulmonary function test, St. George's Respiratory Questionnaire (SGRQ), 6-minute walking (6MW) distance, exacerbations, transthoracic echocardiographic measurements (ejection fraction and right ventricular pressure [RVP]), and chest CT measurements (airway index, air trapping, and emphysema index) were analyzed for their relationships with the mPA, Ao, and mPA/Ao ratio. Exacerbation was defined as aggravation of at least 1/3 respiratory symptoms (dyspnea, cough, or sputum purulence) for ≥2 days that required an unscheduled visit to hospital or emergency room, or hospitalization for additional treatment. Patients were interviewed at the clinic every 3 months with the pre-structured interview sheet including the question if they have visited clinics or emergency rooms due to increased sputum amount, purulent sputum, or deterioration of dyspnea within 3months. Exacerbation documented in the hospital where the patients were enrolled, was traced by medical records. If the patients visited other clinics or other emergency rooms, we collected the name of clinics or emergency rooms, reason for visit, visit date, and frequency through the pre-structured interview sheet.

### Chest CT measurements

Volumetric CT scans were performed on all patients in the beginning of the enrollment during stable disease status at full inspiration and expiration using a 16-multi detector CT scanner (Somatom Sensation; Siemens Medical System, Erlangen, Germany). Two radiologists independently measured the mPA and Ao. The mPA and Ao at the level of bifurcation were used to calculate the mPA/Ao ratio. mPA was determined by the widest diameter perpendicular to the long axis of the main pulmonary artery at the level of pulmonary artery bifurcation. The Ao was determined by the transverse diameter of the aorta at the level of pulmonary artery bifurcation. The κ value of the intraclass correlation coefficient was 0.95 (95% confidence interval (CI), 0.93–0.96) for mPA, and 0.94 (95% CI, 0.92–0.95) for Ao.

For the airway index, air trapping, and emphysema index, images of the whole lung were extracted automatically and the attenuation coefficient of each pixel was calculated. The cut-off level between normal lung density and low-attenuation areas was defined as -950 Hounsfield units.[14] The volume fraction of the lung below -950 Hounsfield units was calculated automatically at full inspiration, and termed the emphysema index. The mean lung density was calculated automatically during expiration and inspiration. The air-trapping index was estimated by calculating the ratio of the mean lung density at expiration and inspiration. Airway dimensions were measured near the origin of the two segmental bronchi (RB1, LB1+2). The airway dimensions (wall area, lumen area, and wall area percent [15]) were measured in each bronchus. The airway index (wall area percent) was defined as wall area/ (wall area + lumen area) × 100.[16]

### Statistical analysis

Statistical analysis was performed using SAS 9.3 (SAS Institute Inc., Cary, NC, USA). The correlations between clinical characteristics and the mPA, Ao, and mPA/Ao ratio were analyzed. Multivariate linear regression analyses adjusted for age, body surface area (BSA), lung function, and SGRQ were performed to evaluate the effects of mPA, Ao, and the mPA/Ao ratio on the 6MW distance. We defined patients with an mPA/Ao ratio >0.8 and ≤0.8 as the high and low groups, respectively. An mPA/Ao ratio of >0.8 was considered to indicate the presence of relative pulmonary artery enlargement, as this represents the top 10 percentile in the distribution. The baseline characteristics are summarized using percentages to describe categorical variables, and the two groups (mPA/Ao ratio ≤0.8 vs. >0.8) were compared using chi-square analyses. Continuous variables are presented as median (interquartile range), as assessed by the Mann-Whitney nonparametric *U* test. Differences in COPD exacerbation during the follow-up period were analyzed with logistic regression analysis for repeated measures using Generalized Estimating Equations. The risk of exacerbation is described as the odds ratio (OR) with 95% confidence interval (CI). In all analyses, *P-*values <0.05 were deemed statistically significant.

## Results

### Baseline characteristics of study population

The mean age was 65.8 years, and 219 (96.9%) patients were male. The FEV_1_, FVC, and FEV_1_/FVC were 1.64 L, 3.46 L, and 47.3%, respectively. According to the previous Global initiative for Chronic Obstructive Lung Disease criteria, 15.9%, 53.1%, 26.1%, and 4.9% of patients were categorized as mild, moderate, severe, and very severe, respectively. The mPA and Ao were 23.7 and 36.4 mm, respectively, and the mPA/Ao ratio was 0.66 (Table 1). None of the patients showed an mPA/Ao ratio >1.

**Table 1:** Baseline characteristics of the patients^a^.

Baseline characteristics	Total
	(n = 226)
Age, years	65.8 ± 7.5
Male sex	219 (96.9)
BMI, kg/m^2^	23.3 ± 3.3
BSA, m^2^	1.70 ± 0.15
Smoking amount, pack-years	46.7 ± 24.9
Current smoker	72 (31.9)
Underlying diseases	
Congestive heart failure	4 (1.8)
Myocardial infarction	4 (1.8)
Hypertension	59 (26.1)
Diabetes mellitus	23 (10.2)
Asthma	56 (24.8)
Pulmonary function tests	
FEV_1_, L	1.64 ± 0.56
FEV_1_, % predicted	61.2 ± 19.1
FVC, L	3.46 ± 0.80
FVC, % predicted	91.3 ± 18.3
FEV_1_/FVC, %	47.3 ± 11.0
DLco, % predicted (n = 221)	80.7 ± 25.1
RV, % predicted (n = 213)	147.1 ± 57.6
TLC, % predicted (n = 208)	117.5 ± 20.5
RV/TLC, % (n = 208)	47.9 ± 12.9
SGRQ, total score	33.4 ± 17.6
6MW distance, m (n = 217)	447.0 ± 74.9
Oxygen saturation, %	96.3
Exacerbation in previous year	41 (18.1)
Chest computed tomography parameters	
mPA, mm	23.7 ± 3.3
Ao, mm	36.4 ± 3.6
mPA/Ao ratio	0.66 ± 0.09
Airway index (n = 221)	66.1 ± 6.3
Air trapping, % (n = 221)	94.4 ± 3.4
Emphysema index (n = 221)	22.8 ± 15.2
Echocardiography	
Ejection fraction, % (n = 62)	61.3 ± 5.7
Right ventricular pressure, cmH_2_O (n = 107)	29.0 ± 7.9

^a^ Data are presented as numbers (percentages) for categorical variables. Continuous variables are presented as means ± standard deviations.

BMI, body mass index; BSA, body-surface area; FEV_1_, forced expiratory volume in 1s; FVC, forced vital capacity; DLco, diffusion capacity of carbon monoxide; RV, residual volume; TLC, total lung capacity; SGRQ, St. George's Respiratory Questionnaire; 6MW, 6-minute walking; mPA, main pulmonary artery diameter; Ao, aorta diameter.

### Clinical characteristics/CT correlations

Table 2 demonstrates the correlations between the clinical characteristics and the mPA, Ao, and mPA/Ao ratio. Age and BSA positively associated with mPA (G = 0.154, *P* = 0.02; G = 0.154, *P* = 0.02) and Ao (G = 0.257, *P*<0.001; G = 0.228, *P* = 0.001). The 6MW distance negatively associated with the mPA (G = -0.124, *P* = 0.034) and mPA/Ao ratio (G = -0.133, *P* = 0.025). Moreover, the RVP on echocardiography positively associated with the mPA (G = 0.327, *P* = 0.001) and mPA/Ao ratio (G = 0.323, *P* = 0.001).

**Table 2:** Correlations between clinical characteristics and chest CT parameters.

Clinical characteristics	mPA		Ao		mPA/Ao ratio	
	G	*P*-value	G	*P*-value	G	*P*-value
Age, years	0.154	0.02	0.257	<0.001	-0.033	0.623
BMI, kg/m^2^	0.162	0.015	0.141	0.034	0.061	0.359
BSA, m^2^	0.154	0.02	0.228	0.001	-0.006	0.931
Pulmonary function tests						
FEV_1_, L	-0.016	0.245	0.014	0.416	-0.045	0.249
FEV_1_, % predicted	-0.014	0.416	0.017	0.4	-0.018	0.395
FVC, L	-0.083	0.107	-0.043	0.26	-0.047	0.239
FVC, % predicted	-0.049	0.234	-0.109	0.05	0.031	0.324
FEV_1_/FVC, %	0.005	0.469	0.067	0.157	-0.031	0.32
DLco, % predicted (n = 221)	0.008	0.453	0.06	0.186	-0.035	0.304
RV, % predicted (n = 213)	-0.014	0.423	-0.09	0.099	0.041	0.28
TLC, % predicted (n = 208)	0.004	0.48	-0.008	0.455	0.002	0.491
RV/TLC, % (n = 208)	0.063	0.184	0.107	0.061	-0.024	0.364
SGRQ, total score	0.012	0.427	-0.34	0.304	0.035	0.3
6MW distance, m (n = 217)	-0.124	0.034	0.032	0.322	-0.133	0.025
Oxygen saturation, %	-0.032	0.642	-0.085	0.211	0.022	0.753
Chest computed tomography parameters						
Airway index	0.01	0.443	-0.006	0.467	0.013	0.423
Air trapping	-0.007	0.459	0.055	0.208	-0.053	0.215
Emphysema index	-0.055	0.207	-0.189	0.002	0.077	0.127
Echocardiography^b^						
Ejection fraction, % (n = 62)	-0.101	0.434	-0.086	0.508	0.002	0.986
Right ventricular pressure, cmH_2_O (n = 107)	0.327	0.001	0.001	0.998	0.323	0.001

CT, computed tomography; mPA, main pulmonary artery diameter; Ao, aorta diameter; BMI, body mass index; BSA, body surface area; FEV1, forced expiratory volume in 1s; FVC, forced vital capacity; DLco, diffusion capacity of carbon monoxide; RV, residual volume; TLC, total lung capacity; SGRQ, St George's Respiratory Questionnaire; 6MW, 6-minute walking.

### Multivariate analysis for 6MW distance

Table 3 shows the multivariate linear analyses to determine the relationships of the mPA, Ao, and mPA/Ao with the 6MW distance. After adjustment for age, BSA, FEV_1_% predicted, FEV_1_/FVC, and SGRQ total score, the mPA/Ao ratio was significantly associated with 6MW distance (*β* = -107.7, *P* = 0.017). In all three models, age, BSA, and SGRQ were significantly related with the 6MW distance.

**Table 3:** Multivariate linear analyses for the effects of clinical characteristics, including chest CT parameters, on the 6MW distance (n = 217).

Clinical characteristics	*β*	*P*-value	95% CI
**Model 1**			
Age, years	-2.915	< 0.001	-1.08 –-1.75
BSA, m^2^	88.524	0.006	25.16–151.88
FEV_1_, % predicted	.138	0.694	-0.55–0.83
FEV_1_/FVC, %	.311	0.604	-0.87–1.49
SGRQ, total score	-1.388	< 0.001	-1.91 –-0.87
mPA, mm	-2.427	0.065	-5.00–0.15
**Model 2**			
Age, years	-3.322	< 0.001	-4.53 –-2.11
BSA, m^2^	65.785	0.047	0.80–130.77
FEV_1_, % predicted	.194	0.583	-0.50–0.89
FEV_1_/FVC, %	.289	0.632	-0.89–1.47
SGRQ, total score	-1.403	< 0.001	-1.93 –-0.88
Ao, mm	1.469	0.245	-1.02–3.95
**Model 3**			
Age, years	-3.162	< 0.001	-4.31 –-2.02
BSA, m^2^	76.917	0.015	15.19–138.65
FEV_1_, % predicted	.181	0.603	-0.51–0.87
FEV_1_/FVC, %	.262	0.66	-0.91–1.43
SGRQ, total score	-1.389	< 0.001	-1.91 –-0.87
mPA/Ao ratio	-107.717	0.017	-195.69 –-19.74

CT, computed tomography; 6MW, 6-minute walking; CI, confidence interval; BSA, body-surface area; FEV_1_, forced expiratory volume in 1s; FVC, forced vital capacity; SGRQ, St. George's Respiratory Questionnaire; mPA, main pulmonary artery diameter; Ao, aorta diameter.

#### mPA/Ao ratio ≤ vs. > 0.8

Among the 226 COPD patients, 210 (92.9%) and 16 (7.1%) were classified as low (mPA/Ao ratio ≤0.8) and high (mPA/Ao ratio >0.8) groups, respectively (Table 4). The age, proportion of males, and BSA did not differ between the groups. The amount of smoking was higher (44 *vs*. 32.5 pack-years, *P* = 0.024), congestive heart failure and myocardial infarction were more common, and diabetes mellitus and asthma were less common in the low group. Other clinical characteristics of COPD did not differ between the two groups. The mPA and Ao were larger in the high group (28.4 *vs*. 23.3 mm, *P*<0.001 and 36.4 *vs*. 34.2 mm, *P* = 0.008, respectively), as was the RVP (34.0 *vs*. 27.9 mmH_2_O, *P* = 0.029).

**Table 4:** Clinical characteristics of patients with mPA/Ao ratio≤0.8 and mPA/Ao ratio>0.8^a^.

Clinical characteristics	mPA/Ao ratio ≤ 0.8	mPA/Ao ratio > 0.8	*P*-value
	(n = 210)	(n = 16)	
Age, years	67.0 (61.0–70.0)	65.5 (60.1–68.8)	0.629
Male sex	204 (97.1)	15 (93.8)	0.450
BMI, kg/m^2^	23.4 (21.0–25.4)	23.7 (22.1–25.8)	0.346
BSA, m^2^	1.71 (1.61–1.80)	1.70 (1.64–1.76)	0.954
Smoking history, pack-years	44.0 (29.8–55.5)	32.5 (21.4–40.5)	0.024
Current smoker	69 (32.9)	3 (18.8)	0.479
Underlying disease			
Congestive heart failure	4 (1.9)	0 (0)	0.001
Myocardial infarction	4 (1.9)	0 (0)	0.004
Hypertension	54 (25.7)	5 (31.2)	0.831
Diabetes mellitus	21 (10.4)	2 (12.5)	0.013
Asthma	50 (24.9)	6 (37.5)	0.024
Tuberculosis	37 (18.4)	2 (12.5)	0.836
Gastroesophageal reflux disease	3 (1.5)	0 (0)	0.183
Pulmonary function tests			
FEV_1_, L	1.6 (1.2–2.0)	1.7 (1.1–1.9)	0.835
FEV_1_, % predicted	59.5 (47.0–76.0)	62.5 (47.0–68.7)	0.861
FVC, L	3.4 (2.9–4.0)	3.1 (2.8–3.9)	0.398
FVC, % predicted	90.5 (78.0–103.3)	89.0 (75.8–103.8)	0.793
FEV_1_/FVC, %	48.0 (40.0–56.0)	47.0 (40.2–56.8)	0.896
Severity of airway obstruction^b^			0.510
Mild	35 (16.7)	1 (6.3)	
Moderate	110 (52.4)	10 (62.5)	
Severe or very severe	65 (30.9)	5 (31.3)	
SGRQ, total score	29.6 (20.3–46.6)	36.6 (22.4–41.2)	0.511
6MW distance, m	454.5 (400.0–497.5)	427.5 (405.0–467.5)	0.254
Oxygen saturation, %	97.0 (95.0–98.0)	96.0 (95.3–97.0)	0.693
Exacerbation in previous year	36 (17.1)	5 (31.3)	0.359
CT			
mPA, mm	23.3 (21.6–25.4)	28.4 (26.7–29.6)	<0.001
Ao, mm	36.4 (33.8–39.1)	34.2 (32.1–35.6)	0.008
Airway index	66.2 (62.3–69.6)	66.9 (65.6–69.9)	0.226
Air trapping	94.8 (92.7–96.7)	95.2 (91.7–96.7)	0.733
Emphysema index	20.1 (9.9–34.4)	21.7 (7.1–39.2)	0.790
Echocardiography			
Ejection fraction, %^c^	62.0 (59.0–64.0)	62.0 (60.0–68.5)	0.300
Right ventricular pressure, cmH_2_O^d^	27.9 (23.0–33.0)	34.0 (29.3–41.6)	0.029

^a^ Data are presented as numbers (percentages) for categorical variables. Continuous variables are presented as median (interquartile range).

^b^ The severity of airway obstruction was based upon the percent of predicted FEV_1_ in accordance with the previous GOLD criteria (FEV_1_ ≥80% predicted, mild; FEV_1_ = 50~79% predicted, moderate; FEV_1_ = 30~49% predicted, severe; FEV_1_ <30% predicted, very severe).

^c^ Echocardiography data were available in 57 patients with PA:A ratio ≤0.8 and in 5 patients with PA:A ratio **>**0.8.

^d^ Echocardiography data were available in 98 patients with PA:A ratio ≤0.8 and in 9 patients with PA:A ratio **>**0.8.

mPA, main pulmonary artery diameter; Ao, aorta diameter; BMI, body mass index; BSA, body surface area; CT, computed tomography; SGRQ, St. George's Respiratory Questionnaire; 6MW, 6-minute walking.

### Associations between mPA/Ao and exacerbations

Logistic regression analyses to identify clinical factors associated with exacerbation of COPD revealed that an mPA/Ao ratio >0.8 correlated with exacerbations at the 1-year (OR = 3.51, 95% CI 1.37–9.02, *P* = 0.009) and 3-year follow-ups (OR = 2.1, 95% CI 1.22–3.56, *P* = 0.007) (Table 5). Exacerbation history in the previous year was also related with exacerbations at the 1-year (OR = 2.12, 95% CI 1.27–3.52, *P* = 0.004) and 3-year follow-ups (OR = 2.04, 95% CI 1.42–2.90, *P*<0.001). Conversely, the SGRQ score showed inverse relationships with COPD exacerbation at both follow-ups.

**Table 5:** Clinical factors associated with COPD exacerbations in the KOLD cohort.

Clinical factors	1-year follow-up^a^			3-year follow-up^b^		
	OR	95% CI	*P*-value	OR	95% CI	*P* value
Exacerbation in previous year	2.12	1.27–3.52	0.004	2.04	1.42–2.90	<0.001
FEV_1_, % predicted, 1-percentage increase	1.01	0.99–1.02	0.222	1	0.99–1.01	0.432
SGRQ, 1-point increase	0.99	0.97–1.00	0.035	0.98	0.97–0.99	<0.001
Age, 1-year increase	0.99	0.96–1.02	0.523	0.99	0.97–1.02	0.508
mPA/Ao ratio, >0.8	3.51	1.37–9.02	0.009	2.1	1.22–3.56	0.007

^a^ n = 205

^b^ n = 171

COPD, chronic obstructive pulmonary disease; KOLD, Korean obstructive lung disease; OR, odds ratio; CI, confidence interval; FEV_1_, forced expiratory volume in 1s; SGRQ, St. George's Respiratory Questionnaire; mPA, main pulmonary artery diameter; Ao, aorta diameter.

## Discussion

Herein, we found that the mPA/Ao ratio independently correlated with exercise capacity (6MW distance) and that an mPA/Ao ratio >0.8 was an independent risk factor for exacerbation at the 1-year and 3-year follow-ups in Korean COPD patients. Moreover, RVP related with the mPA/Ao ratio, while the pulmonary function, airway index, air trapping, and emphysema index did not.

The prevalence of PH in COPD patients ranges between 30–70%, and often develops in advanced COPD.[17] COPD-associated PH has significant clinical implications such as functional limitations and poor prognosis, with a 5-year survival rate of 20–36%.[17–19] Severe PH, defined as mean pulmonary arterial pressure (mPAP) >40 mmHg, can also present in mild-to-moderate COPD patients, who show worse mortality than those with mild-to-moderate PH (mPAP, 20–40 mmHg).[8] PH is disproportionate to the degree of airflow limitation, because pulmonary vascular changes can appear at the early stages of disease and in smokers without airflow limitation.[15,20] An experimental study with animal models of COPD revealed that changes in the pulmonary vessels occurred prior to development of emphysema,[21] and PH observed in non-severe COPD is considered the combined result of systolic and diastolic left ventricular failure, inflammation, and other comorbid conditions.[22,23]

CT is a useful, non-invasive tool for evaluating both intrinsic lung disease and intrathoracic vasculature in COPD patients. Recently, extensive researches on the usefulness of CT in COPD have been conducted regarding the coronary artery calcium score, lung cancer screening, and pulmonary emphysema subtyping.[24–26] However, these evaluations may need specialized software and expertise, while the mPA/Ao ratio is readily measurable by chest CT and requires minimal training with good inter-observer and intra-observer agreements.[11,13,27,28] The two radiologists in our study also showed excellent agreements with k value of more than 0.9. Moreover, the mPA/Ao ratio has been shown to correlate with mPAP (γ = 0.74, *P*<0.001) and to suggest diverse clinical conditions.[29,30] The sensitivity and specificity of the mPA/Ao ratio >1 to predict mPAP >25 mmHg have been reported as 68.4–96.0% and 59.0–92.0%, respectively, in various population.[11,22,30–32] According to the report of Iyer et al., mPA/Ao ratio >1 showed sensitivity of 73% and specificity of 84% for identifying resting PH in patients with COPD.[11] In this study, we furthermore demonstrated a significant, albeit weaker correlation than in previous reports, between the RVP and mPA/Ao ratio (γ = 0.323, *P* = 0.001) in COPD patients.[13] Unfortunately, assessment of the predictive value of mPA/Ao ratio >1.0 for PH was not possible, because no patient showed such elevation.

In the COPDGene study, relative pulmonary artery enlargement (mPA/Ao ratio>1) was a useful biomarker for future exacerbation events in COPD.[13] Patients with an mPA/Ao ratio >1 group were more frequently female; had higher body mass index (BMI), worse airflow obstruction, and more common supplemental oxygen use; and showed higher prevalence of congestive heart failure and thromboembolic disease. On the other hand, Iyer et al. reported a higher prevalence of congestive heart failure in patients with an mPA/Ao ratio >1, but no difference in airflow obstruction.[11] In our study, the mPA/Ao >0.8 group less frequently showed cardiac diseases such as congestive heart failure and myocardial infarction, but more frequently experienced diabetes mellitus or asthma. However, because of the small number of cardiac diseases, no firm conclusions could be drawn. Moreover, the lung function and other quantitative assessments on chest CT did not differ between the groups and did not correlate with the mPA, Ao, or mPA/Ao ratio. This finding is in accordance with previous reports that lung function has no utility in the prediction of mPAP in COPD.[23,33,34]

Besides the relationship of the mPA/Ao ratio with hemodynamics, we investigated the prognostic and functional predictive roles of the mPA/Ao ratio in COPD. Wells et al. reported an mPA/Ao ratio >1 as a significant predictor of severe exacerbation in the COPDGene and Evaluation of COPD Longitudinally to Identify Predictive Surrogate Endpoints cohorts during 3 years of follow-up.[13] Herein, we provided further evidence of the prognostic role of elevated mPA/Ao ratio, even at a subclinical level (>0.8), for COPD exacerbation in Korea.

Subclinical levels of mPA/Ao in Korean COPD may have shown clinical implication because our cohort study population had relatively lower distribution of mPa/Ao ratio. This distinct distribution can be explained by several clinical characteristics in our study population. BMI and BSA were relatively lower in our study (BMI: 23.3 kg/m^2^ and BSA: 1.7m^2^) compared with those in other studies (BMI: 25–28 kg/m^2^ and BSA: 1.8–1.9 m^2^) of various diseases.[11,27,28,35–38] Wells et al. showed that COPD patients with mPA/Ao >1 had higher BMI (29.7 ± 7 kg/m^2^).[13] Moreover, according to COPD stages, proportion of supplemental oxygen use and comorbidities, the severity of disease was lower in our study population.[13,39,40] More patients in our study were categorized into mild-moderate group more (69%) and less into very severe group (4.9%) than those in COPDGene study (52.9% and 15.6%) and ECLIPSE study (44.2% and 13.7%). Moreover, proportion of supplemental oxygen use was higher in COPDGene study (27%) while none used it in our study.[13] For the comorbidities, number of patients with hypertension (50%) and congestive heart failure (5–7%) was higher in other studies than our study (26% and 2%).[13,39] Lastly, different ethnicities are also important factors. While our study population consisted of only Koreans, the proportion of Asians was none or small in other studies. Therefore, subclinical levels of mPA/Ao may reveal clinical importance in different population.

Although the mechanisms of the relationship between exacerbation and increased mPA/Ao ratio requires clarification, repetition of subclinical exacerbation associated with transient increases in the mPAP could lead to permanent vascular remodeling, which consequently may cause future exacerbation and poor prognosis.[23,41] Moreover, vascular remodeling may lower the threshold of hemodynamic stability and make the patients vulnerable to exacerbation.

Finally, among the limited data on the relationship between PH and exercise function in COPD, Sims et al. determined that higher systolic pulmonary arterial pressure was independently associated with reduced exercise function in severe COPD.[6] This is the first study to demonstrate that a higher mPA/Ao ratio was associated with significantly lower exercise impairment, that is, a shorter 6MW distance, independent of demographic factors and lung function (Table 3). Wells et al. also reported that the 6MW distance negatively correlated with the mPA/Ao ratio (G = -0.49, *P* = 0.02).[37] However, on multivariate logistic regression analysis, as the 6MW distance decreased by 1 foot, the OR for mPA/Ao ratio >1 was 1 (95% CI 1.00–1.01). Pulmonary vascular remodeling in COPD may result in ventilation–perfusion mismatch, leading to worsening of hypoxemia and failure of the pulmonary vascular bed to accommodate the increased cardiac output during exercise.[6] Further, early right ventricular changes in non-severe COPD are associated with lower exercise tolerance.[35]

The strengths of our study are that it was the first study to determine the functional and prognostic impacts of the mPA/Ao ratio, which is easily measurable on chest CT, using the KOLD cohort. However, there were some limitations. First, the number of included patients was relatively small, even though this was a multicenter observational study. Second, over half the patients showed a moderate degree of airflow obstruction, so our finding might not be applicable to patients with severe airway obstruction. However, in contrast to the previous studies on COPD patients with severe severity, we have demonstrated clinical significance of mPA/Ao ratio in relation to exacerbation and exercise capacity in COPD patients with less severity. Third, the mean mPA/Ao ratio was relatively low compared to in previous studies. The mPA and Ao independently correlate with age and body size, leading to decreases in the in mPA/Ao ratio with aging and lower body size as compared with the general population and COPD patients of other ethnicities.[27,29,42] Fourth, 0.8 was used as the cut-off of the mPA/Ao ratio and was the best values to predict acute exacerbation of Korean COPD patients; however, its clinical utility for individual patients should be taken with caution. Lastly, information on nocturnal symptoms or symptoms of sleep apnea was not collected in the cohort. Obstructive sleep apnea is related with higher PA pressure and PH is a poor prognostic sign in patients with obstructive sleep apnea.[43]

## Conclusion

Herein, the functional and prognostic implications of the mPA/Ao ratio were evaluated in the KOLD cohort. The mPA/Ao ratio was an independent negative predictor of functional capacity, measured by the 6MW distance. This finding may help predict the exercise capacity in COPD patients unable to fully perform exercise tolerance tests for various reasons. Moreover, we showed that an mPA/Ao ratio >0.8 was a significant predictor of COPD exacerbation at 1 and 3 years. Nevertheless, the role of mPA/Ao for monitoring the therapeutic response requires further study.
